# The SPFH complex HflK-HflC regulates aerobic respiration in bacteria

**DOI:** 10.1371/journal.pbio.3003077

**Published:** 2025-04-07

**Authors:** María Isabel Pérez-López, Paul Lubrano, Georgia Angelidou, Sarah Hoch, Timo Glatter, Nicole Paczia, Hannes Link, Victor Sourjik

**Affiliations:** 1 Max Planck Institute for Terrestrial Microbiology, Marburg, Germany; 2 Center for Synthetic Microbiology (SYNMIKRO), Marburg, Germany; 3 University of Tübingen,Tübingen, Germany; Interfaculty Institute of Microbiology and Infection Medicine, Massachusetts Institute of Technology, Howard Hughes Medical Institute, UNITED STATES OF AMERICA

## Abstract

The bacterial HflK-HflC membrane complex is a member of the highly conserved family of SPFH proteins, which are present in all domains of life and include eukaryotic stomatins, flotillins, and prohibitins. These proteins organize cell membranes and are involved in various processes. However, the exact physiological functions of most bacterial SPFH proteins remain unclear. Here, we report that the HflK-HflC complex in *Escherichia coli* is required for growth under high aeration. The absence of this complex causes a growth defect at high oxygen levels due to a reduced abundance of IspG, an essential iron-sulfur cluster enzyme in the isoprenoid biosynthetic pathway. This reduction might be related to lower stability of IspG and several other proteins, including the iron siderophore transporter TonB, in the absence of the HflK-HflC complex. Our results suggest that decreased IspG activity leads to lower levels of ubiquinone and misregulated expression of multiple respiratory enzymes, including cytochrome oxidases, and consequently reduced respiration and lower ATP levels. This impact of the *hflK hflC* deletion on aerobic respiration resembles the mitochondrial respiratory defects caused by the inactivation of prohibitins in mammalian and yeast cells, indicating functional parallels between these bacterial and eukaryotic SPFH proteins.

## Abstract 2

The bacterial HflK-HflC membrane complex, part of the highly conserved SPFH protein family, plays a key role in cell membrane organization. This study shows that HflK-HflC is important for aerobic respiration in bacteria, where it stabilizes proteins like IspG and TonB; reduced IspG activity impairs respiration and energy production.

## Introduction

Members of the SPFH (Stomatin, Prohibitin, Flotillins, and HflK-HflC) protein family have been identified in all three domains of life [1,2]. A common feature of these membrane proteins is an evolutionarily conserved prohibitin homology (PHB) domain (also called SPFH domain), which may have lipid–protein binding properties [3]. The SPFH proteins share a common property of self-oligomerization into large membrane-spanning or membrane-anchored complexes, and they appear to have diverse but poorly understood functions, mostly related to the organization of lipid membranes [4–6].

In eukaryotic cells, SPFH proteins are present at various cellular locations, including the plasma membrane, Golgi apparatus, mitochondria, and endoplasmic reticulum [3,7], where they play an important role in scaffolding proteins and specific lipids within lipid domains. The SPFH proteins are involved in various biological processes, with stomatins contributing to the regulation of ion channels [8,9], and flotillins being associated with signal transduction, endocytosis, and neuronal regeneration [7,10,11]. Prohibitins, located in the inner mitochondrial membrane, form large hetero-oligomers that interact with the AAA+ membrane protease [12]. The absence of prohibitins affects several cellular processes, including cell proliferation, apoptosis, and respiration, but the mechanisms behind these effects remain largely unknown [13–16].

Bacterial SPFH family proteins were described more than two decades ago [1], but their functions are even less understood than those of their eukaryotic counterparts. Research on Gram-positive bacteria has revealed certain structural and functional similarities between eukaryotic and bacterial flotillins [17], with the scaffolding activity of these bacterial flotillins being important for the regulation of membrane fluidity and the assembly of protein complexes involved in signal transduction [18–20]. Even less is known about the functions of SPFH proteins in gram-negative bacteria. In *Escherichia coli*, four proteins containing the PHB domain have been identified: QmcA, YqiK, and the complex HflK-HflC (=HflKC), all of which are localized in the inner membrane. While the functions of QmcA and YqiK remain unclear, the HflKC complex is known to interact with FtsH, an integral membrane ATP-dependent Zn^2 +^ metalloprotease belonging to the AAA+ family of ATPases [21]. HflK and HflC have a similar secondary structure consisting of a single transmembrane helix at the N-terminus followed by large periplasmic SPFH1 and SPFH2 domains and coiled-coil domains. HflK, HflC, and FtsH form a large complex consisting of 12 copies of the HflKC heterodimer, providing a large compartmentalized cage for 4 embedded FtsH hexamers [22,23]. This complex shares features with the multimeric assemblies formed by eukaryotic prohibitins in the inner membrane of mitochondria that interact with a hexameric AAA+ protease homologous to FtsH [24,25].

FtsH degrades membrane and cytoplasmic proteins involved in several cellular pathways [26–28], and deletion of the *ftsH* gene causes a severe growth defect [29]. In contrast, no pronounced growth phenotype has been reported for *E. coli* lacking the HflKC complex [30], and the physiological significance of this complex, including the HflKC-dependent regulation of FtsH, remains unclear [28]. Here, we demonstrate that the HflKC complex is important for growth under conditions of high aeration. This effect could be explained by a decrease in the abundance of IspG, a key iron-sulfur cluster enzyme in the isoprenoid biosynthesis pathway, that leads to reduced levels of ubiquinone, which is essential for aerobic respiration. Our results suggest that low levels of ubiquinone lead to the misregulation of cytochrome oxidases and other respiratory enzymes, likely mediated by the ArcAB two-component system, and to the reduced respiration. Although the mechanisms responsible for lowering IspG levels in the absence of the HflKC complex remain to be elucidated, we demonstrate that the effect of *hflKC* deletion depends on FtsH and that stability of IspG and of several other proteins is significantly reduced in this background. These findings reveal a novel function of the HflKC complex in aerobic respiration, which may be analogous to the function of eukaryotic prohibitins in mitochondria.

## Results

### HflKC complex is important for *E. coli* growth under high aeration

**Fig 1:**
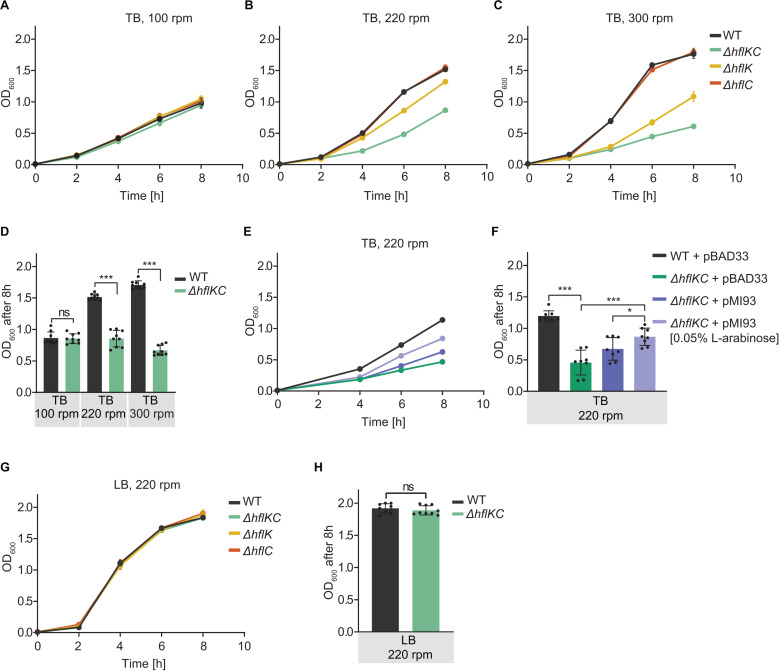
HflKC complex is important for *E. coli* growth under high aeration. **(A–D)** Growth of *E. coli* Δ*hflKC*, Δ*hflK*, and Δ*hflC* strains and corresponding WT in TB medium at 100 rpm **(A)**, 220 rpm **(B)**, or 300 rpm **(C)** shaking rate, quantified by optical density at 600 nm (OD_600_), and the final OD_600_ after 8 h of growth **(D)**. **(E, F)** Growth of *ΔhflKC* and WT strains carrying either an empty vector (pBAD33) or the pBAD33-derived expression plasmid pMI93 encoding *hflK* and *hflC*, in TB at 220 rpm **(E)** and corresponding final OD_600_ after 8 h of growth **(F)**. Where indicated, 0.05% L-arabinose was added to induce expression. **(G, H)** Growth of *E. coli ΔhflK*, *ΔhflC*, *ΔhflKC*, and WT strains in LB at 220 rpm **(G)** and corresponding final OD_600_ after 8 h of growth **(H)**. For these and other growth curves, the data represent the mean and standard deviation (SD) of three independent cultures grown in the same representative experiment. Whenever not visible, error bars are smaller than the symbol size. See S1A–S1D Fig for additional biological replicates. For final OD_600_ comparisons, the data represent the mean and SD of independent cultures, indicated by dots, grown in three different experiments. Significance of indicated differences between samples: *p < 0.05, ***p < 0.001, and ns = not significant by unpaired *t*-test. All data underlying this figure can be found in S1 Data.

When an *E. coli* strain deleted for the *hfl* genes was phenotyped under various conditions, it exhibited a growth defect that was dependent on aeration and medium composition. For *E. coli* grown in rich tryptone broth (TB) medium on an orbital shaker, the culture density of strains deleted for the *hflK* and *hflC* genes was similar to that of the wild-type (WT) strain at low shaking rates (Figs 1A and S1A). However, at higher shaking rates, the growth of the *ΔhflK ΔhflC* (= *ΔhflK*C) strain was significantly slower than that of the WT strain (Figs 1B, 1C, 1D, S1B, and S1C). While WT growth increased at higher shaking rates, as expected from better aeration, growth of the *ΔhflKC* mutant even decreased. In agreement with the optical density measurements, the number of the colony-forming units (CFUs) also decreased (S1D Fig). The observed growth defect of the *ΔhflKC* strain was specific, as it could be largely complemented by co-expressing the *hflK* and *hflC* genes from a plasmid (Fig 1E and 1F). Surprisingly, although HflK and HflC are known to form a heterodimeric complex, only the deletion of *hflK* caused the growth phenotype that was similar to that of the strain lacking both genes (Figs 1A–1C and S1A–S1C). In contrast, growth of the *ΔhflC* strain did not differ from that of the wild type. Nevertheless, the deletion of *hflC* had some impact in the background of the *hflK* deletion, since the growth defect of the *ΔhflKC* strain was more pronounced than that of the *ΔhflK* strain.

Interestingly, no growth defect was observed for the *ΔhflKC* strain at high aeration in an even richer Luria-Bertani (LB) medium (Figs 1G, 1H, and S1E), which contains yeast extract in addition to tryptone and NaCl that are present in both LB and TB. Consistently, the addition of yeast extract to TB resulted in a dose-dependent reduction in the difference between growth of the WT and *ΔhflKC* strains (S1F Fig). Thus, the absence of the HflKC complex causes a specific aeration- and medium-dependent growth phenotype but not a general growth defect. Moreover, microscopy images showed no apparent differences in morphology between the WT and *ΔhflKC* cells (S1G Fig).

We next tested whether the addition of a fermentable carbon source to TB could restore the growth of the *ΔhflKC* mutant. However, while supplementation of TB with glucose resulted in faster growth, the difference between the *ΔhflKC* and the WT strains remained (S1H and S1I Fig). The growth phenotype of the *ΔhflKC* strain further remained evident when cells were cultured at high aeration in M9 minimal medium containing glucose as the sole carbon source (S1J and S1K Fig). Consistent with the dependence of the growth defect observed for the *ΔhflKC* strain on aeration, no difference in growth from the wild type was observed in TB under anaerobic conditions (S1L and S1M Fig).

### Absence of the HflKC complex affects the abundance of respiration-related proteins

**Fig 2:**
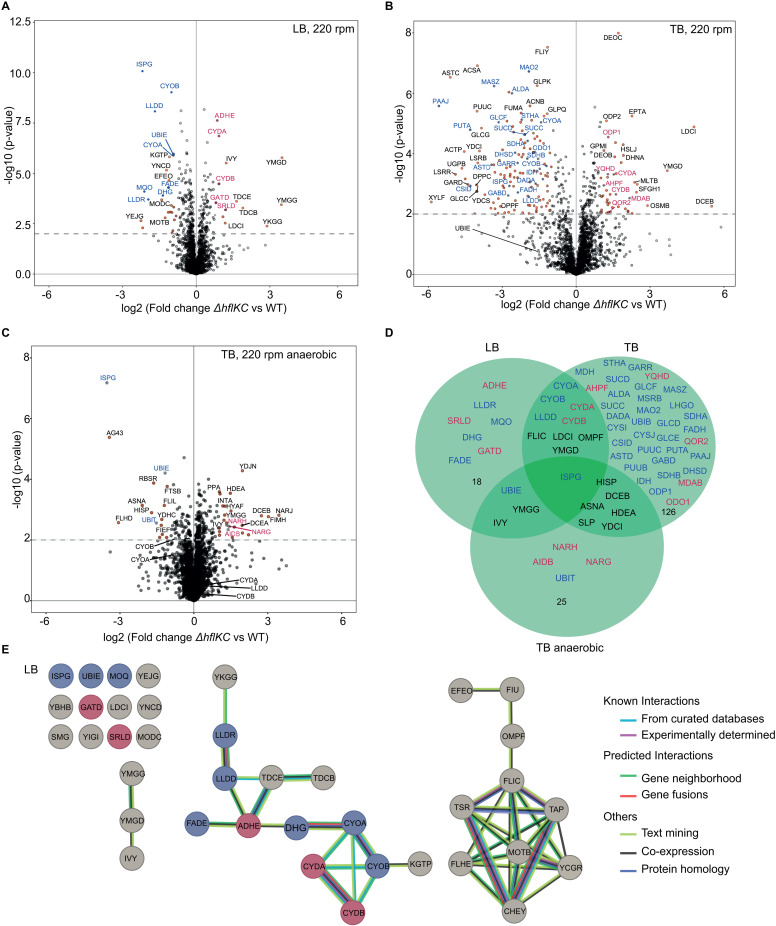
Absence of HflKC complex affects the abundance of respiration-related and other proteins. **(A–C)** Difference in protein levels between *ΔhflKC* and WT strains. Cultures were grown in LB **(A)**, TB **(B)**, or anaerobically in TB **(C)**. Data represent six (LB) or three (TB) independent cultures. Proteins with differences in expression that were considered significant (see Tables 1 and S1–S3) are labeled, with respiration-related proteins highlighted in either blue (downregulated) or red (upregulated). The underlying data can be found in S2 Data. **(D)** Commonalities and differences between proteins significantly up- or down-regulated in *ΔhflKC* under different conditions. Colors of protein labels are the same as in other panels. Respiration-related proteins and those affected under more than one condition are shown, and the number of other proteins affected under a particular condition is shown. **(E)** The STRING diagram showing proteins that are significantly up- or down-regulated in the *ΔhflKC* deletion strain. Links indicate specified types of relationships between proteins, with the interaction score confidence threshold of 0.4. Proteins related to respiration are colored in red (upregulated) or blue (downregulated).

To identify possible causes of the observed growth defect, we first analyzed changes in whole-cell protein levels caused by the deletion of *hflK* and *hflC* genes, for *E. coli* cultures grown either in LB or in TB under strong shaking. Consistent with similar growth of the *ΔhflKC* and WT strains in LB (Fig 1G), only a small number of proteins showed pronounced differences in abundance under these conditions (Fig 2A and Tables 1 and S1). In contrast, differences between cultures grown in TB, where the deletion strain showed a growth defect at high aeration (Fig 1B), were much more extensive (Fig 2B and S2 Table). Fewer differences in protein composition were observed when the two strains were grown under anaerobic conditions (Fig 2C and S3 Table), consistent with their similar growth (S1L Fig).

**Table 1:** Respiratory proteins showing significant differences between *ΔhflKC, ΔhflK,* or *ΔhflC* and WT strains during growth in LB at 220 rpm.

		Log_2_ (fold change)		
Protein	Function	*ΔhflKC* vs. WT	*ΔhflK* vs. WT	*ΔhflC* vs. WT
ISPG	Oxidoreductase involved in isoprenoid biosynthesis	−2.28	−2.37	0.02
MQO	Malate: quinone oxidoreductase	−2.15	−1.84	−0.33
LLDR	L-lactate dehydrogenase operon regulator	−1.90	−2.26	−0.44
LLDD	L-lactate dehydrogenase	−1.66	−1.52	−0.38
DHG	Quinoprotein glucose dehydrogenase	−1.34	−1.26	−0.23
FADE	Acyl-CoA dehydrogenase	−1.13	−0.50	−0.78
CYOB	Cytochrome bo(3) ubiquinol oxidase subunit 1	−1.01	−1.01	−0.20
UBIE	Ubiquinone biosynthesis	−0.92	−0.93	−0.01
CYOA	Cytochrome bo(3) ubiquinol oxidase subunit 2	−0.91	−0.85	0.00
SRLD	Sorbitol-6-phosphate 2-dehydrogenase	1.18	1.44	−0.36
CYDA	Cytochrome bd-I ubiquinol oxidase subunit 1	0.96	0.85	−0.03
ADHE	Fused acetaldehyde-CoA dehydrogenase	0.94	0.89	−0.08
CYDB	Cytochrome bd-I ubiquinol oxidase subunit 2	0.91	0.68	−0.05
GATD	Galactitol-1-phosphate 5-dehydrogenase	0.82	1.12	0.28

Despite this dependence on incubation conditions, the levels of several proteins showed consistent differences between the *ΔhflKC* and WT strains (Fig 2D). Among the proteins whose abundance was significantly perturbed under aerobic conditions in both LB and TB were two cytochrome quinol oxidases, CyoABCD (*bo*_*3*_) and CydAB (*bd*), which are used by *E. coli* under aerobic (i.e., high O_2_) or microaerobic (low O_2_) conditions, respectively [31]. The levels of these two cytochrome quinol oxidases showed opposite changes, with the catalytic subunits CyoAB of the aerobic quinol oxidase *bo*_*3*_ being reduced in the *ΔhflKC* strain, and the levels of the microaerobic quinol oxidase CydAB being elevated. The expression of several other respiration-related proteins was also affected in LB (Fig 2E and S1 Table), and even more so in TB under aerobic conditions (S2 Table).

**Fig 3:**
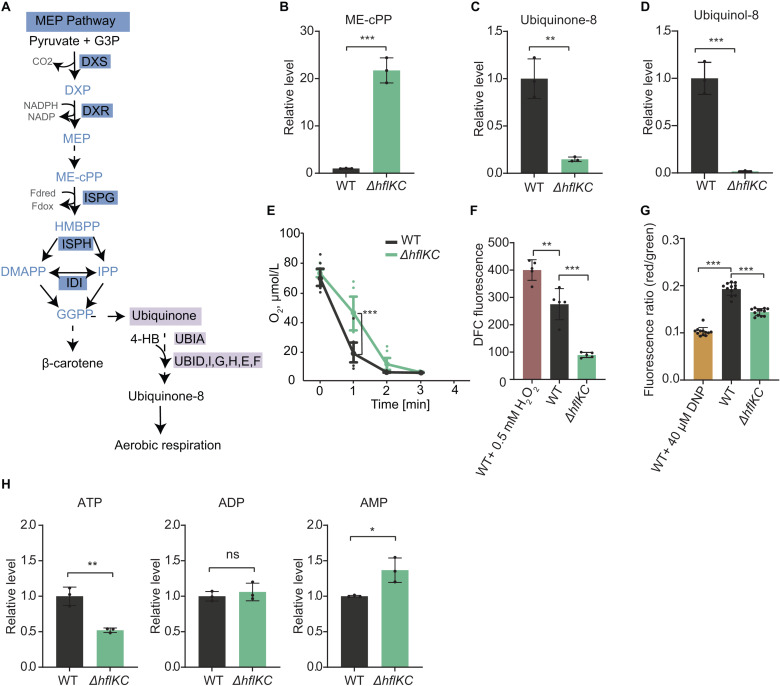
*ΔhflKC* strain shows reduced ubiquinone levels, aerobic respiration, and ATP levels. **(A)** MEP pathway in *E. coli.* Metabolic intermediates are colored in light blue, and selected enzymes are shown on either dark blue (MEP pathway) or purple (ubiquinone biosynthesis) background. DXP: 1-deoxy-D-xylulose 5-phosphate; MEP: 2-C-methyl-D-erythritol 4-phosphate; ME-cPP: 2-C-methyl-D-erythritol 2,4-cyclic diphosphate; HMBPP: 1-hydroxy-2-methyl-2-(E)-butenyl 4-diphosphate; DMAPP: dimethylallyl diphosphate; IPP: isopentenyl diphosphate; and GGPP: geranylgeranyl diphosphate; 4-HB: 4-hydroxybenzoate. **(B–D)** Levels of the IspG substrate ME-cPP **(B)** and of ubiquinone-8 **(C)** and ubiquinol-8 **(D)** in *ΔhflKC* relative to the WT strain. Strains grown at 220 rpm in either M9 glucose minimal medium **(B)** or in TB **(C, D)**. The data represent the mean and SD of three independent cultures. **(E)** Oxygen consumption by WT and *ΔhflKC* cells. The cultures were grown in TB at 220 rpm and resuspended in fresh TB, and changes in the levels of dissolved oxygen were quantified over time. Large symbols represent the mean and SD of eight independent measurements (shown by small dots) for cells from one culture. See also S4 Fig. **(F)** Levels of ROS in WT and *ΔhflKC* cells grown in TB at 220 rpm, measured using the DCF fluorescent probe as illustrated in S5 Fig. Treatment with hydrogen peroxide (H_2_O_2_) was used as a positive control for elevated ROS levels. The data represent the mean and SD of five measurements with 30,000 cells per measurement. **(G)** Membrane potential of WT and *ΔhflKC* cells grown in TB at 220 rpm, measured using the DiOC_2_(3) dye as illustrated in S6 Fig. DNP was used as a control. The data represent the mean and SD of 12 measurements from 2 independent experiments with 30,000 cells per measurement. (H) Levels of ATP, ADP, and AMP (H) in cells grown in M9 glucose minimal medium at 220 rpm. Means of three independent cultures and SD are shown. Significance of indicated differences between samples: *p < 0.05, **p < 0.01, ***p < 0.001, and ns = not significant by unpaired *t*-test. All data underlying this figure can be found in S3 Data.

We also observed a strong reduction in the levels of two metabolic enzymes, UbiE and IspG, which are involved in the biosynthesis of respiratory chain electron carriers. UbiE methyltransferase is part of the ubiquinone and menaquinone biosynthetic pathway [32]. IspG belongs to the methylerythritol phosphate (MEP) pathway and catalyzes the conversion of ME-cPP (2C-methyl-D-erythritol 2,4-cyclodiphosphate) to HMBPP (hydroxymethylbutenyl 4-diphosphate), a key substrate for the production of isoprenoids, which are also required for quinone biosynthesis [33] (Fig 3A). The decreased abundance of these two enzymes was observed even under anaerobic conditions and thus independent of the respiratory status of *E. coli* cells. Notably, although the change in UbiE level was below the significance threshold in TB under aerobic conditions, its expression was nevertheless reduced (Fig 2B). In contrast, the levels of most other respiratory proteins, including cytochrome oxidases, showed no significant differences between the anaerobically grown *ΔhflKC* and WT cultures (Fig 2C and S3 Table). In addition to the cluster of respiration-related proteins, significant changes in the levels of other proteins were observed in the *ΔhflKC* strain, too. In particular, proteins involved in motility and chemotaxis were downregulated in LB (Fig 2E and S1 Table) and also in TB under both aerobic and anaerobic conditions (S2 and S3 Tables).

The abundances of most known FtsH substrates [34] or of FtsH itself were not significantly affected in either LB or TB (S2A and S2B Fig), confirming that the *ΔhflKC* deletion does not lead to a general change in the FtsH activity. Surprisingly, no significant change in the abundance could be observed for SecY that was previously suggested to be an HflKC-dependent FtsH substrate [35]. Of note, another established HflKC-dependent substrate of FtsH, the phage lambda protein CII [36,37], is not present in our *E. coli* strain. The level of one known FtsH substrate, a lipopolysaccharide biosynthesis enzyme LpxC [38], was modestly elevated in TB (S2B Fig), indicating that the HflKC complex might promote its degradation by FtsH. However, all of the most prominently affected proteins were not among the established FtsH substrates.

We therefore tested whether the observed impact of the *hflKC* deletion on the proteome composition depends on FtsH. Although FtsH is normally essential, viable *ftsH* knockouts carrying suppressor mutations have been described [21,39]. We compared the proteome composition of these previously published isogenic *ftsH* deletion strains with or without the *hflKC* knockout [21]. Of note, these strains also carry deletion of *qmcA* that encodes another SPFH protein, to avoid possible interference between these two systems [21]. These experiments were performed in LB, because changes in protein levels observed upon the *hflKC* deletion in the WT background were more specific in this medium compared to TB (Fig 2D). In the *ftsH* background, the *hflKC* deletion had no impact on the expression on IspG or other respiration-related proteins (S2C Fig), suggesting that it is FtsH-dependent.

Although our primary focus was on the phenotype of the strain lacking the entire HflKC complex, we also evaluated the individual effects of the *hflK* and *hflC* deletions on the proteome composition. Consistent with similarity of their growth phenotypes, the proteome profiles of the *ΔhflKC* and *ΔhflK* strains were similar (Figs 2A, 2B, S3A, and S3C, and Table 1). In contrast, the *ΔhflC* strain showed little change in proteome composition compared to the wild type, despite having a reduced level of HflK (S3B and S3D Fig). Thus, the growth phenotype and changes in the proteome observed in the *ΔhflKC* strains are primarily due to the absence of HflK, whereas the lack of HflC can be tolerated by the cell, which suggests a difference in the functionality between HflK and HflC (see Discussion).

Data from Figs 2A, S3A, and S3B. Differences with *p* < 0.05 and log2 of fold change > 0.8 were considered significant.

### *ΔhflKC* strain shows reduced ubiquinone levels, aerobic respiration, and ATP levels

Given the greatly reduced levels of IspG in the *ΔhflKC* strain and the importance of the MEP pathway for the ubiquinone biosynthesis (Fig 3A), we examined the impact of the *ΔhflKC* deletion on the MEP pathway and on ubiquinone levels. Consistent with the expected low net IspG activity, the level of the IspG substrate ME-cPP was largely elevated in the *ΔhflKC* strain compared to the wild type (Fig 3B), whereas the levels of the oxidized (ubiquinone-8) and especially of the reduced (ubiquinol-8) forms of ubiquinone were strongly decreased (Fig 3C and 3D). Thus, the downregulation of IspG, and possibly also of UbiE downstream in the pathway (Fig 3A), apparently causes a disruption in the ubiquinone biosynthesis in the absence of the HflKC complex.

Because low levels of ubiquinone, along with the downregulation of the aerobic quinol oxidase *bo*_*3*_, could cause a reduction in the aerobic respiratory activity, we compared the consumption of dissolved oxygen by the *ΔhflKC* and WT cell cultures. Indeed, oxygen consumption by the *ΔhflKC* culture was significantly lower (Figs 3E and S4). Further consistent with the reduced respiration, the level of reactive oxygen species (ROS) assessed using the dichlorodihydrofluorescein (DCF) probe (Figs 3F and S5), as well as the membrane potential (MP) assessed using the 3,3′-diethyloxacarbocyanine iodide DiOC_2_(3) probe (Figs 3G and S6) were also lower in *ΔhflKC* cells.

Such reduced respiration and the resulting decrease in the MP could lead to lower ATP production in *ΔhflKC* cells. This decrease was indeed evident when the levels of ATP, ADP, and AMP were quantified in the *ΔhflKC* and WT cultures using targeted metabolomics. We observed that the level of ATP was lower and the level of AMP was higher in *ΔhflKC* cells, whereas the level of ADP remained unchanged (Fig 3H).

### Reduced levels of IspG account for the respiratory phenotype of the *ΔhflKC* strain

**Fig 4:**
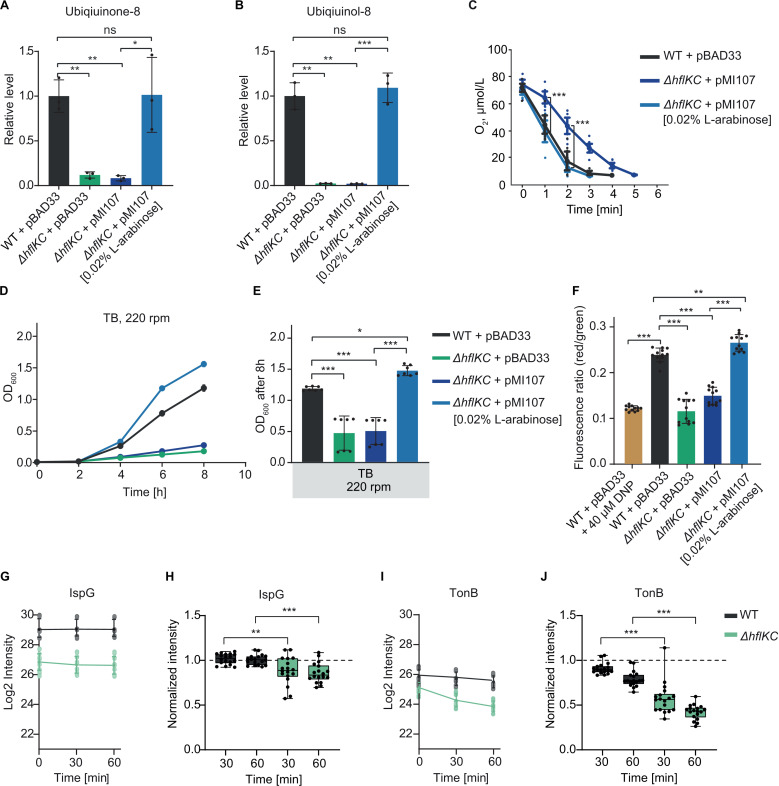
Reduced IspG levels cause the respiratory phenotype of the ***ΔhflKC* strain.** **(A, B)** Levels of ubiquinone-8 **(A)** and ubiquinol-8 **(B)** in the *ΔhflKC* strain, expressing IspG from an inducible plasmid vector, relative to the WT strain carrying pBAD33. The WT or *ΔhflKC* strains, transformed with empty vector pBAD33 or with pMI107 encoding *ispG* were grown in TB at 220 rpm; 0.02% L-arabinose was added to induce expression where indicated. The data represent the mean and SD of three independent cultures. **(C)** Oxygen consumption by the indicated strains. Measurements were performed as in Fig 3E. Large symbols represent the mean and SD of eight independent measurements for cells from one culture. See also S7A Fig. **(D, E)** Growth of the indicated strains **(D)** and corresponding final OD_600_ after 8 h of growth **(E)**. The data in **(D)** represent the mean and SD of three independent cultures grown in the same representative experiment. The data in **(E)** represent the mean and SD of seven independent cultures, indicated by dots, grown in three different experiments. **(F)** Measurements of MP in the indicated strains, performed using the DiOC_2_(3) dye as in Fig 3G. Significance of indicated differences between samples: *p < 0.05, **p < 0.01, ***p < 0.001, and ns = not significant by unpaired *t*-test. **(G, H)** Abundance of IspG determined by proteomics in the WT or *ΔhflKC* cultures in LB at 220 rpm at indicated times after the inhibition of translation, represented as log_2_ protein intensity **(G)**, and the same data normalized to the initial time point for each independent culture and plotted on a linear scale **(H)**. **(I, J)** Abundance of TonB, determined and presented as in panels **(G, H)**. The data in **(G–J)** represent the mean and SD of 18 independent cultures, measured in 3 different experiments with 6 cultures each. Significance of indicated differences between samples: **p < 0.01, ***p < 0.001, and ns = not significant by unpaired *t*-test. All data underlying this figure can be found in S4 Data.

Collectively, our data suggest that the lower ubiquinone levels, misregulation of respiratory enzymes, and consequently reduced aerobic respiration and poor growth at high aeration, may be due to low levels of IspG and/or UbiE. Since the reduction in IspG abundance was more pronounced and consistent across data sets, and because of its upstream position in the metabolic network leading to ubiquinone production, we hypothesized that low levels of IspG might be the primary cause of the observed respiratory phenotype. Supporting this hypothesis, induced expression of IspG from a plasmid restored ubiquinone (Fig 4A) and ubiquinol (Fig 4B) levels in *ΔhflKC* cells, as well as their oxygen consumption (Figs 4C and S7A), to WT levels. Growth of the *ΔhflKC* strain at high aeration (Fig 4D and 4E) and cell MP (Fig 4F) also increased upon the induction of IspG expression, even exceeding the WT levels.

These results support our hypothesis that low levels of IspG are directly or indirectly responsible for all observed respiration-related phenotypes of the *ΔhflKC* strain. To assess the effects of the reduced IspG level, and because *ispG* is essential in *E. coli*, we used the dCas9 *ispG* knockdown. This knockdown had no effect on *E. coli* growth at low aeration (S7B Fig), but reduced growth at high aeration (S7C, S7D, and S7E Fig), effectively phenocopying the impact of *ΔhflKC* deletion. Furthermore, microscopic images showed no morphological differences between the control strain and induced dCas9 for *ispG* knockdown (S7F Fig). Moreover, we observed changes in the abundance of multiple respiration-related proteins in the *ispG* knockdown (S7G Fig), including reduced levels of CyoAB and increased levels of CydAB (S7H Fig and S4 Table), further supporting the causal connection between the downregulation of IspG and the misregulation of cytochrome oxidases and other respiratory enzymes. In contrast, the levels of motility-related and some other proteins were not affected by *ispG* knockdown, suggesting that their changes in the *ΔhflKC* strain are unrelated to the reduced IspG levels.

To better understand possible origin of the reduced IspG abundance in the *ΔhflKC* strain, we first tested the levels of *ispG* transcript. This comparison revealed no significant difference between the *ΔhflKC* and WT strains (S8A Fig and S7 Table), suggesting that transcriptional regulation is unlikely to be the cause of the reduced IspG levels. An alternative explanation could be the increased degradation of IspG in the absence of the HflKC complex. To investigate this, we next performed proteome-wide comparison of protein stability between the WT and *ΔhflKC* cultures, by quantifying changes in protein levels over time upon addition of the translation inhibitor chloramphenicol. While no reduction in the level of IspG was observed in the WT cells, there was a modest but significant decrease of the IspG stability in the *ΔhflKC* strain (Fig 4G and 4H), which can at least partly account for the reduced levels of IspG in this background.

We then expanded our analysis to identify other proteins with lower stability in the absence of the HflKC complex. The most prominent reduction in stability was observed for the cytoplasmic membrane protein TonB (Fig 4I and 4J) that is involved in the uptake of iron siderophores [40]. Other proteins with decreased stability (S8A Fig) included the membrane protein MreC involved in the peptidoglycan biosynthesis and the cytoplasmic enzyme UbiF (Fig 3A). The latter is immediately downstream of UbiE in the ubiquinone biosynthesis pathway, and we hypothesize that the decreased stability of UbiF might be caused by the reduced level of its substrate. Interestingly, two stress-response proteins, RpoS and RMF, showed strongly reduced stability but elevated initial protein levels, indicating a complex feedback interplay between their increased degradation and upregulation of their expression in the *ΔhflKC* background.

Given increased degradation of TonB and the requirement of the iron-sulfur cluster for IspG activity, we examined whether iron limitation could increase growth defect of the *ΔhflKC* strain. While growth of the wild type was not affected by addition of a low (20 µM) concentration of iron chelator deferoxamine (DFO), the *ΔhflKC* mutant indeed displayed a significant reduction in growth under the same conditions (S9 Fig).

### Changes in the abundance of respiratory proteins are caused by activation of the ArcAB system

Finally, we aimed to investigate the mechanism responsible for the global changes in the abundance of respiratory proteins which are caused by the reduced level of IspG and likely contribute to the respiratory defect of the *ΔhflKC* strain. In *E. coli*, the levels of (oxidized) quinones are known to repress the two-component ArcAB system [41]. The latter, in turn, controls the expression of a large number of respiration-related genes to mediate the transition from aerobic to anaerobic growth [42,43]. Thus, we hypothesized that the reduced ubiquinone biosynthesis in the *ΔhflKC* strain, due to low IspG activity, might cause activation of the ArcAB system, leading to downregulation of aerobic respiratory genes and induction of the microaerobic cytochrome oxidase *bd-*I.

Indeed, although deletion of the sensory kinase gene *arcB* itself negatively affects growth, we observed no additional impact of the *hflKC* gene deletion in the *ΔarcB* background on aerobic growth in TB (S10A–S10D Fig). Furthermore, the changes in proteome composition caused by the *arcB* deletion were largely opposite to those caused by the *hflKC* deletion (S10E and S10F Fig and S5 Table), and the levels of CyoAB or CydAB proteins exhibited no significant differences when comparing *ΔarcB* and *ΔhflKC ΔarcB* strains (S10G and S10H Fig and S6 Table). This is consistent with our hypothesis that changes in the levels of respiratory proteins in the *ΔhflKC* strain are dependent on the ArcAB system (S11 Fig). In contrast, the downregulation of IspG and UbiE, as well as of several other proteins, including those involved in motility, appears to be independent of the ArcAB system. Notably, levels of the *arcB* transcript or of the ArcA and ArcB proteins themselves were not affected by the *hflKC* deletion (S10I and S10J Fig), confirming that it is the activity of the ArcAB system that is influenced by the reduction of IspG levels.

## Discussion

Although SPFH proteins are conserved between prokaryotes and eukaryotes, suggesting their fundamental importance for cellular function, the specific roles of these proteins remain poorly understood [2,44]. Particularly in prokaryotes, only a few examples of the functional importance of SPFH proteins have been reported [45–48]. Studies of SPFH proteins in *E. coli* have so far identified mild phenotypes that have not been mechanistically explained [30,49]. This is particularly surprising for the HflKC complex, which is known to form a large oligomeric inner membrane cage that encloses the nearly-essential AAA-type protease FtsH [22,23] and is thought to regulate FtsH access to some of its substrates [23]. However, its only previously well-established phenotype was repression of the FtsH-mediated proteolysis of the phage lambda protein CII [36,37].

Here we demonstrate that the HflKC complex plays an important role during growth of *E. coli* under conditions of high aeration. Our results suggest that the growth defect of the *ΔhflKC* strain at high oxygen levels is directly or indirectly caused the reduced abundance of IspG, an enzyme in the MEP pathway for isoprenoid biosynthesis (S11 Fig). The MEP pathway provides essential precursors for several cellular processes [50], including the biosynthesis of pigments and ubiquinone [51–53]. The level of ubiquinone-8 was indeed greatly reduced in *ΔhflKC* cells. Besides limiting the precursor supply for ubiquinone biosynthesis, and possibly as a consequence of such precursor limitation, the low level of IspG causes the downregulation of UbiE. This could also explain the reduced levels and decreased stability of other enzymes in the ubiquinone biosynthesis pathway observed under some of our experimental conditions. In turn, this decrease in the ubiquinone-8 biosynthesis appears to lead to the reduction of aerobic respiration in *ΔhflKC* cells, likely due to a combination of lower activity of cytochrome ubiquinol oxidases and the perturbed expression of multiple respiratory enzymes.

The observed IspG-dependent changes in the levels of respiration-related proteins in the *ΔhflKC* strain prominently include downregulation of the major *E. coli* cytochrome ubiquinol oxidase *bo*_*3*_ (CyoABCD), which operates under high O_2_ conditions, and upregulation of the less efficient cytochrome ubiquinol oxidase *bd* (and CydAB), which is normally used under microaerobic conditions [31], and they could be largely explained by the activation of the two-component system ArcAB. This system allows bacteria to adapt to changes in oxygen availability and it activates the expression of genes involved in anaerobic respiration while inhibiting the expression of aerobic respiratory genes [42]. Its sensory kinase, ArcB, is normally repressed at high O_2_ by oxidized ubiquinone [41,54], but this repression appears to be alleviated in *ΔhflKC* cells due to the overall reduction in the ubiquinone levels, causing an aberrant activation of the ArcAB system. However, the levels of IspG and UbiE were affected by the *ΔhflKC* deletion even in the absence of ArcB, confirming that the ArcAB system is downstream of these proteins in the regulatory cascade (S11 Fig).

In contrast to TB or M9 glucose minimal medium, no growth defect at high aeration was observed for *ΔhflKC* cells in LB. Compared to TB, changes in the levels of respiration-related proteins in LB were also limited to a smaller set of proteins, including IspG, UbiE, and both cytochrome oxidases. Although the causes of these differences in growth and protein expression require further investigation, the effect was due to the presence of yeast extract. Possible explanations include the availability of metabolic intermediates that partially complement the impact of IspG and UbiE downregulation on respiratory activity, or alternatively lesser importance of respiration for *E. coli* growth in the presence of metabolites contained in yeast extract. In either case, the observed medium- and aeration-specificity of the *ΔhflKC* phenotype suggests that the lack of HflKC does not have a general impact on *E. coli* growth or morphology.

The molecular mechanisms behind reduction of the IspG levels in the absence of the HflKC complex need to be elucidated, too, but our data suggest that this effect directly or indirectly depends on the activity of FtsH and could be related to the significantly decreased stability of IspG in the *ΔhflKC* background. Nevertheless, it remains to be seen whether this modest reduction in stability is sufficient to fully explain the steady-state difference in protein levels, and also whether the reduced stability is due to a direct degradation of IspG by FtsH. Alternatively, downregulation of IspG might be caused by the prominent destabilization of TonB observed in the absence of the HflKC complex, which might lower the uptake of iron required by the iron-sulfur cluster protein IspG (S11 Fig).

In addition to the changes in the levels of multiple respiration-related proteins, the absence of the HflKC complex also directly or indirectly affected levels and stability of a number of other proteins, including those involved in *E. coli* motility and stress response. Two prominently destabilized proteins, TonB and MreC, reside in the inner membrane, potentially consistent with the hypothesis that the HflKC complex might protect membrane proteins from the FtsH activity [23]. However, such deprotection must be protein-specific as it was not observed for the other, known membrane protein substrates of FtsH.

Interestingly, although the FtsH-regulatory HflKC complex is normally a heterodimer that contains equal number of HflK and HflC subunits [22], we observed a striking asymmetry in the effects of individual deletions of the *hflK* and *hflC* genes. While the loss of *hflK* causes the growth phenotype and changes in the proteome similar to the absence of the entire HflKC complex, the deletion of the *hflC* gene alone has no apparent effect and only slightly enhances the phenotype of the *hflK* deletion. This observation is even more surprising considering that the deletion of *hflC* causes a decrease in the level of HflK, as is frequently the case for the unassembled components of the heterooligomeric complexes. This implies that HflK alone, even at reduced protein levels, can largely carry out the function of the HflKC complex. Possibly consistent with that, although the overall structures of HflK and HflC are similar, HflK has an additional C-terminal extension that resides inside the HflKC complex and interacts with FtsH, indicating that HflK may be more important for the assembly of the HflKC-FtsH complex and for the FtsH regulation [22,23].

Although HflK and HflC are phylogenetically distant from eukaryotic prohibitins PHB1 and PHB2, the PHB1-PHB2 complex in the mitochondrial membrane also forms a ring-like heterooligomer that regulates the activity of the AFG3L2 AAA + metalloprotease homologous to FtsH [22,24,25]. Notably, prohibitins have been similarly associated with different aspects of respiratory activity. For instance, the knockdown of PHB1 reduces the activity of the respiratory complex in human mitochondria [15], while PHB2 has been implicated in the regulation of assembly of the respiratory complex IV [55,56]. Moreover, prohibitin was shown to interact with the complex IV subunits to prevent their proteolysis by m-AAA protease in yeast cells [57]. Our results demonstrate a different mode of regulation of respiratory activity by the bacterial analog of this complex, through control of the ubiquinone biosynthesis. This mechanism is unlikely to have immediate relevance for eukaryotes, given differences between the quinone biosynthesis and iron uptake pathways between eukaryotes and bacteria, but it is evolutionary intriguing that the HflKC and PHB1-PHB2 complexes are not only structurally but also functionally similar.

## Materials and methods

### Bacterial strains, plasmids, and growth conditions

*Escherichia coli* K-12 MG1655 [58] was used as the WT strain in this study. *ΔhflK* and *ΔhflC* gene deletions were constructed using P1 transduction from the Keio collection strains (JW 4132 and JW 4133, respectively). *ΔhflKC, ΔhflKC ΔarcB,* and *ΔarcB* strains were constructed using lambda red recombination as described previously [59]. Kanamycin cassettes were flipped out using FLP-FLP recombination target (FRT) recombination [60]. All knockout constructs were verified by polymerase chain reaction (PCR). *E. coli* YYdCas9 derived from *E. coli* K-12 (BW25993) was used as a background strain to construct *ispG* knockdown as described previously [61]. Plasmid expression vectors carrying *hflK-hflC* and *ispG* genes were constructed by amplifying DNA fragments from the MG1655 genome by PCR using Q5 high-fidelity DNA polymerase and cloned into pBAD33 [62] using Gibson assembly [63]. All strains and plasmids are listed in the S8 Table.

Strains were grown in LB medium (10-g tryptone, 10-g NaCl, and 5-g yeast extract per liter), TB medium (10-g tryptone and 5-g NaCl per liter), TB supplemented with 0.4% of glucose or M9 minimal medium with glucose as sole carbon source (5 g/L). M9 medium was composed by (per liter): 7.52 g Na_2_HPO_4_ 2H_2_O, 5 g KH_2_PO_4_, 1.5 g (NH_4_)2SO_4_, 0.5 g NaCl. The following components were sterilized separately and then added (per liter of final medium): 1 mL 0.1 M CaCl_2_, 1 mL 1 M MgSO_4_, 0.6 mL 0.1 M FeCl_3_, 2 mL 1.4 mM thiamine HCl, and 10 mL trace salts solution. The trace salts solution contained (per liter): 180 mg ZnSO_4_ 7H_2_O, 120 mg CuCl_2_ 2H_2_O, 120 mg MnSO_4_ H_2_O and 180 mg CoCl_2_ 6H_2_O. Antibiotics (kanamycin 50 µg/mL, ampicillin 100 µg/mL, chloramphenicol 34 µg/mL) and inducers of expression were added where necessary.

For all measurements, OD of overnight cultures were adjusted to OD_600_ of 4 in 1 mL, and consequently diluted 1:100 in 50 mL fresh media and grown in 100 mL flasks at 37 °C on an orbital shaker at indicated shaking rates (100, 220, or 300 rpm). For the iron chelator, a final concentration of 20-µM DFO was added to LB media. For anaerobic growth, sealed flasks where oxygen was replaced with nitrogen were used. For the quantification of CFU, three independent cultures of the strains were grown in TB for 4 h and subsequently serially diluted in phosphate-buffered saline (PBS) (80 g/L NaCl, 2 g/L KCl, 2 g/L KH_2_PO_4_, 11.5 g/L Na_2_HPO_4_, pH 7.4), and 100 µL of the diluted cultures were plated on LB agar plates, between 50 and 200 colonies were counted manually, and the CFU titer was calculated per mL of the original culture.

### Construction of the ispG knockdown

Different protospacers designed along *ispG* gene were cloned in the plasmid vector pgRNA [64]. Plasmids were then transformed into *E. coli* YYdCas9. Expression of dCas9 was induced with 0.2-µM aTC (anhydrotetracycline). Knockdown efficiency was validated using growth measurements, as IspG is an essential enzyme and the decrease in its level causes a defect in growth. Among the tested protospacers, the sequence with the strongest effect on growth inhibition (AATTCCTGACGCGAACAGGT; pMI112) was selected for further experiments.

### Total cell proteomics

Cultures were grown as described above until OD_600_ of 0.4 under aerobic and 0.15 under anaerobic conditions. Biomass was adjusted to OD_600_ =  3 in 1 mL to have an equal amount of cells per sample. Pellets were washed twice with ice-cold PBS and stored at −80 °C.

For protein extraction, cell pellets were dissolved in 300 µL of 2% sodium-lauroyl sarcosinate (SLS) and 100-mM ammonium bicarbonate. Cells were lysed by incubation at 90 °C for 15 min and subsequent sonication (Vial Tweeter, Hielscher) with 80% amplitude for 30 s. Cell lysates were reduced by adding 5 mM (final concentration) Tris(2-caboxyethyl)phosphine and incubating at 95 °C for 15 min followed by alkylation (10-mM iodoacetamide final concentration, 30 min at 25 °C).

The amount of extracted proteins was measured using BCA protein assay (Thermo Fisher Scientific). Fifty microgram total protein was then digested with 1-µg trypsin (Promega) overnight at 30 °C in the presence of 0.5% SLS. Following digestion, SLS was precipitated with trifluoroacetic acid (TFA, 1.5% final concentration) and peptides were purified using Chromabond C18 microspin columns (Macherey-Nagel). Acidified peptides were loaded on spin columns equilibrated with 400-µL acetonitrile and then 400-µL 0.15% TFA. After peptide loading, a washing step with 0.15% TFA was performed, followed by elution using 400-µL 50% acetonitrile. Eluted peptides were then dried by vacuum concentrator and reconstituted in 0.15% TFA.

Peptide mixtures were analyzed using liquid chromatography-mass spectrometry using an UltiMate 3000 RSLCnano connected to a Q-Exactive Plus mass spectrometer (both Thermo Scientific) as reported previously [65]. In short, peptides were separated using a gradient from 96% solvent A (0.15% formic acid) and 4% solvent B (99,85% acetonitrile, 0.15% formic acid) to 30% solvent B over 90 or 120 min at a flow rate of 300 nL/min. Mass spectrometry (MS) data were acquired with the following settings: 1 MS scan at a resolution of 70,000 with 50-ms maximum ion injection fill time, and MS/MS at 17,500 scans of the 10 most intense ions with 50-ms maximum fill time. The data were further analyzed using either Progenesis (Waters) or MaxQuant in standard settings [66] using an *E. coli* uniprot database. Follow-up data analysis and data visualization was done with SafeQuant [66] (available under https://github.com/eahrne/SafeQuant), Perseus [67], and Rstudio software. Due to an instrumental upgrade, a part of the total proteome samples were analyzed on an UltiMate 3000 RSLCnano connected to an Exploris 480 (U-Ex) and a Vanquish Neo connected to an Exploris (V-EX). For the U-EX LC peptide separating gradient was reduced to 60 min (6%–35% solvent B for U-EX). For V-EX peptides, they were eluted by an increasing solvent B from 1% to 25% over 45 min and an additional increase to 35% for 15 min. The MS data were acquired in data-independent acquisition mode (DIA) using 45 windows with an isolation window of 14 mz with 1-m/z overlap (see also [68]). MS scan resolution was set to 120,000 (MS1) and 15,000 (DIA) with a scan range of 350–1,400 m/z (MS1) and 320–950 precursor mass range (DIA). Automatic gain control (AGC) target settings were 300% (MS1) and 3,000% (DIA) with a maximum ion injection time of 50 ms (MS1) and 22 ms (DIA). The MS analysis settings for U-Ex and V-Ex were identical.

DIA data were analyzed using DIA-NN version 1.8 [69] and an *E. coli* protein database. Full tryptic digest was allowed with two missed cleavage sites, and oxidized methionines and carbamidomethylated cysteines. Match between runs and remove likely interferences were enabled. The neural network classifier was set to the single-pass mode, and protein inference was based on genes. Quantification strategy was set to any LC (high accuracy). Cross-run normalization was set to RT-dependent. Library generation was set to smart profiling. DIA-NN outputs were further evaluated using SafeQuant and data visualized in Perseus. The proteomics data obtained in this study were deposited at ProteomeXchange under the accession codes PXD051202, PXD058571, and PXD058845.

### Measurement of protein stability

WT and *ΔhflKC* cultures were grown in LB medium at 37 °C and shaking at 220 rpm. Samples were collected at OD_600_ =  0.6, and the biomass was adjusted to OD_600_ =  3 in 1 mL LB. Subsequently, chloramphenicol was added to the final concentration of 200 µg/mL. Samples were collected after 30 and 60 min of incubation at 37 °C and shaking at 220 rpm. All samples were washed twice with PBS, and pellets were stored at −80 °C until proceeding with the protein extraction and analysis by mass spectrometry as described above.

The linear model of protein degradation was fitted for each protein in each biological replicate in R using the standard lm() function. The corresponding code is available at https://doi.org/10.17617/3.KA5MM2. Time, strain and interaction between time and strain were used as predictors, while the protein abundance was treated as predicted variable. Proteins showing significant negative dependence on time and significant interaction between time and strain were classified as having differential protein degradation between the WT strain and the mutant.

### Quantification of ME-cPP and ATP measurements

Cultures were grown in M9 minimal medium supplemented with glucose at 220 rpm. Cells were grown to an OD_600_ =  0.4–0.5, this preculture was used to inoculate cultures at a final volume of 10-mL M9 glucose minimal medium and starting OD_600_ =  0.05, which were allowed to grow until OD_600_ =  0.5. Biomass of OD_600_ =  0.8 was applied on filter disc (PVDF Membranes: 0.45-μm pore size) and immediately transferred into 1-mL acetonitrile: methanol: water (40:40:20 (v/v)) kept at −20 ^o^C. Samples were incubated for 30 min at −20 ^o^C. After that time, 500 µL of the samples were transferred into a 1.5-mL tube at −20 ^o^C and centrifuged at −9 ^o^C and >13.000 rpm for 15 min, and 350 μL of supernatant was transferred to new Eppendorf tubes and stored at −80 °C until LCMS analysis. Fifteen microliter of each sample was mixed with 15 µL of ^13^C-labeled internal standard. Analysis of target metabolites was performed with an Agilent 6495 triple quadrupole mass spectrometer (Agilent Technologies) and an Agilent 1290 Infinity II UHPLC system (Agilent Technologies) as described previously [70]. The temperature of the column oven was 30 °C, and the injection volume was 3 µL. LC solvents A were water with 10-mM ammonium formate and 0.1% formic acid (v/v) (for acidic conditions), and water with 10-mM ammonium carbonate and 0.2% ammonium hydroxide (for basic conditions). LC solvents B were acetonitrile with 0.1% formic acid (v/v) for acidic conditions and acetonitrile without additive for basic conditions. LC columns were an Acquity BEH Amide (30 ×  2.1 mm, 1.7 µm) for acidic conditions and an iHILIC-Fusion(P) (50 ×  2.1 mm, 5 µm) for basic conditions. The gradient for basic and acidic conditions was: 0 min 90% B; 1.3 min 40% B; 1.5 min 40% B; 1.7 min 90% B; and 2 min 90% B. Quantification of metabolite concentrations was based on the ratio of ^12^C and ^13^C peak heights.

### Quantification of ubiquinone-8 and ubiquinol-8

Cultures were grown in TB at 200 rpm until OD_600_ =  0.4–0.8. Biomass was adjusted to OD_600_ =  5 in 1 mL. Cells were collected by centrifugation and washed twice with 1× PBS. Pellet samples were dissolved in a mixture of 150 µL of chloroform, 300 µL of methanol, and 120 µL of water, followed by shaking for 10 min at 4 °C. Afterward, 150 µL of chloroform and 150 µL of 0.85% KCL were added. Samples were centrifuged for 10 min at maximum *g* at 4 °C. The lipid phase was transferred to new tubes and dried out with nitrogen. The relative quantification and annotation of lipids were performed by using HRES-LC-MS/MS. The chromatographic separation was performed using a Acquity Premier CSH C18 column (2.1 ×  100 mm, 1.7-μm particle size, Waters, Milford, USA) a constant flow rate of 0.3 mL/min with mobile phase A being 10-mm ammonium formate in 6:4 acetonitrile: water and phase B being 9:1 isopropanol: acetonitrile (Honeywell, Morristown, New Jersey, USA) at 40 °C. The injection volume was 5 µL. The mobile phase profile consisted of the following steps and linear gradients: 0–1.5 min constant at 37% B; 1.5–4 min from 37% to 45% B; 4–5 min from 45% to 52% B; 5–8 min from 52% to 58% B; 8–11 min from 58% to 66% B; 11–14 min from 66% to 70% B; 11–14 min from 66% to 70% B; 14–18 min from 70% to 75% B; 18–20 min from 75% to 98% B; 20–25 min constant at 98% B; 25–25.1 min from 98% to 37% B; and 25.1–30 min constant at 37% B.

For the measurement, a Thermo Scientific ID-X Orbitrap mass spectrometer was used. Ionisation was performed using a high-temperature electro spray ion source at a static spray voltage of 3,500 V (positive) and a static spray voltage of 2,800 V (negative), Sheath gas at 50 (Arb), auxiliary gas at 10 (Arb), and Ion transfer tube and vaporizer at 325 and 300 °C. Data-dependent MS2 measurement was conducted applying an orbitrap mass resolution of 120,000 using quadrupole isolation in a mass range of 200–2,000 and combining it with a high-energy collision dissociation (HCD). HCD was performed on the 10 most abundant ions per scan with a relative collision energy of 25%. Fragments were detected using the orbitrap mass analyzer at a predefined mass resolution of 15,000. Dynamic exclusion with and exclusion duration of 5 s after 1 scan with a mass tolerance of 10 ppm was used to increase coverage.

Compound Discoverer (CD) 3.3 (Thermo-Fisher Scientific) was used for lipid annotation by matching accurate mass and MS2 spectra against the MS/MS library MS-DIAL LipidBlast (version 68). In addition, two customized in-house libraries were used for the annotation of the target analytes Ubiquinone-8 and Ubiquinol-8, and a set of eight lipids that served as internal standards. For the semi-quantitative comparison of lipid abundance, annotated peaks were integrated using CD 3.3 (Thermo Scientific) and normalization by the default method provided by CD 3.3 and further processed by the statistical tools described elsewhere.

Ubiquinol annotation was done employing CD 3.3 using a customized CD workflow and matching the metabolic features against three different data libraries. The majority of lipids were matched against the MS-Dial LipidBlast library (version68). In addition, two customized in-house libraries were used. The “IS-List.massList” contained the names of the 8 lipids that were used as internal standards (LPE 13:0, PE 40:8, PG 40:8, CL 56:4, Cer 22:1;2, HexCer 26:1;2, and SM 24:1;2) and the “targetedCompounds.massList” contained the ammonium adduct of the ubiquinol-8 and ubiquinone-8 (CoQ8). The library focus for the targeted analytes was created by the in-house MS/MS measured spectra from previous runs and the library focus in the internal standards was created based on the theoretical mass calculated by the elemental formula.

### Measurements of oxygen consumption

Strains were grown in TB at 37 °C and 220 rpm until OD_600_ =  0.4. Biomass was adjusted to an OD_600_ =  1 in 5 mL TB. Cultures were centrifuged and fresh TB medium was added. Samples were transferred to a glass tube that contained an oxygen sensor spot PSt3-YAU-D5-YOP (PresSens, precision sensing). Sample tubes were under vortex for 1 min to achieve maximum oxygenation; then, shaking was stopped and oxygen consumption was measured via the oxygen spot with a fiber optic transmitter.

### Measurement of ROS

The DCF fluorescent probe by Abcam (ab113851 Kit) was used to measure ROS. Strains were grown in TB 37 °C and 220 rpm until OD_600_ =  0.4. Biomass was adjusted to exactly OD_600_ =  0.4 in 1 mL TB. Samples were transferred to a 1.5-mL Eppendorf tube where DCF probe was added to have a final concentration of 20 µM. Samples were gently mixed by inversion, followed by dark incubation for 30 min in the dark at 37 °C. Fluorescence was detected by flow cytometry on a BD LSRFortessa SORP cell analyzer (BD Biosciences, Germany) using a 485-nm laser (80 mW) and a 525/15-nm bandpass filter. Treatment with 0.5 mM of hydrogen peroxide (H_2_O_2_) was used as a positive control for elevated ROS levels.

### Characterization of MP

BacLight Bacterial Membrane Potential kit (B34950 Molecular Probes) was used to measure the MP. Strains were grown in TB at 37 °C and 220 rpm. All samples were diluted in 1× PBS and biomass was adjusted to have OD_600_ =  0.4 in 1 mL. Samples were transferred to a 1.5-mL Eppendorf tube where DiOC_2_(3) was added to a final concentration of 0.03 mM. Samples were gently mixed by inversion, followed by incubation for 15 min at 37 °C in the dark. WT treated with 40 µM of dinitrophenol (DNP) was used as a negative control. Flow cytometry on a BD LSRFortessa SORP cell analyzer (BD Biosciences, Germany) with excitation by a 488-nm laser (100 mW) was used to measure the fluorescence of red (670/30-nm bandpass filter) and green (530/10-nm bandpass filter) channels of DiOC_2_(3). Membrane potential was characterized by the ratio of the red and green fluorescence according to the manufacturer’s instructions.

### Microscopy

The strains were grown in TB and LB media at 37 °C and 220 rpm for 4 h. Subsequently, 3 µL of bacterial cells were spread on a small pad of 1% agarose prepared with PBS. Conventional light microscopy was performed using a Nikon Eclipse *Ti* with an oil immersion objective (100× magnification, 1.45 numerical aperture, Nikon).

### RNA extraction and real-time PCR

Strains were grown in LB medium at 220 rpm until OD_600_ =  0.4 as described above. Cultures were concentrated to have OD_600_ =  1 in 1 mL. After centrifugation pellets were washed twice with cold water and stored at −80 °C. Frozen pellets were resuspended in 800 μL of lysis buffer (2% SDS and 4 mM EDTA) and boiled for 2 min at 90 °C. Subsequently, 800 μL of TRIzol was added and incubated at room temperature for 5 min. To the mixture, 200 μL of phenol:chloroform was added, vortexed for 30 s, and incubated for 10 min. Samples were then centrifuged at 13,000× g and 4 °C for 10 min to separate the phases. The upper aqueous phase containing RNA was transferred to a new tube containing 500 μL of isopropanol for RNA precipitation, which was carried out overnight at −20 °C. The following day, samples were centrifuged at 13,000×*g* and 4 °C for 30 min, and the supernatants were discarded. RNA pellets were washed twice with 70% ethanol, air-dried, and resuspended in 50 μL of nuclease-free water to proceed with DNase treatment. After that, samples were stored at −80 °C.

The real-time PCR (RT-PCR) reactions were performed as described in KAPA SYBR FATS one-step qRT-PCR master mix 2× Kit (KR0393) using 2 µL of 10 ng/μL RNA sample. Primers used for IspG were GTATTTACGTTGGGAATGTGCCG and GATATCAGCGCCAACGCGTTC. Primers for AcrB were CAGTTGGCCGAAGGTTTCCTGCAG and ACTTTCTATCGGTGGTCGTCGAGCAAC. Housekeeping gene ssrA was used as a control with primers ATTCTGGATTCGACGGGATT and AGTTTTCGTCGTTTGCGACT.

## Supporting information

S1 Fig Growth of *ΔhflKC, ΔhflK,* and *ΔhflC* strains under different conditions. (A–C) Growth of E. coli *ΔhflKC*, *ΔhflK*, and *ΔhflC* strains and corresponding WT in TB medium in an orbital shaker at 100 rpm (A), 220 rpm (B), or 300 rpm (C), quantified by optical density at 600 nm (OD_600_). The data represent the mean of three independent cultures that are different from those shown in Fig 1A–1C. Error bars indicate the SD of three independent cultures. **(D)** Colony forming units per mL of WT and *ΔhflKC* grow in TB at 220 rpm. **(E)** Growth of the indicated strains in LB medium in an orbital shaker at 220 rpm. Data represent the mean and SD of three independent cultures, different from the experiment shown in Fig 1G. **(F)** Growth of *E. coli ΔhflKC* and WT strains in TB media at 220 rpm with 20% and 100% of yeast extract (YE), with 100% corresponding to 5 g of YE in 1L of media. The data represent the mean and SD of three independent cultures grown in the same representative experiment. **(G)** Phase-contrast images of WT and *ΔhflKC* from exponential cultures grown in TB or LB, as indicated. Scale bar is 2 µm. **(H, I)** Growth of *ΔhflKC* and WT strains in TB supplemented with 0.4% glucose at 220 rpm (H) and corresponding final OD_600_ after 8 h of growth (I). The data in (H) represent the mean and SD of three independent cultures. **(J, K)** Growth of *ΔhflKC* and WT strains in M9 glucose minimal medium (J) and corresponding final OD_600_ after 8 h of growth (K). The data represent the mean and SD of three independent cultures grown in the same representative experiment. **(L, M)** Growth of *ΔhflKC* and WT strains in TB at 220 rpm under anaerobic conditions (L) and corresponding final OD_600_ after 8 h of growth (M). The data represent the mean and SD of three independent cultures grown in the same representative experiment. Indicated differences between samples: **p < 0.01, ***p < 0.001 or not significant (ns) by unpaired *t* test. All data underlying this Figure can be found in S5 Data.
(EPS)

S2 Fig Comparison of abundances of known FtsH substrates between *ΔhflKC* and WT cells and FtsH dependence of *ΔhflKC* effect on protein levels. **(A, B)** Differences in protein levels between *ΔhflKC* and WT strains grow in LB **(A)** or TB **(B)** at 220 rpm. Data are the same as in Fig 2A and 2B, but with known FtsH substrates and FtsH itself labeled in purple. Data are for six (LB) or three (TB) independent cultures. **(C)** Differences in protein levels between *ΔftsH*::*kan qmcA*::cat *hflKC::tet* and *ΔftsH*::*kan qmcA*::cat strains grow in LB at 30 °C and 220 rpm. Respiratory proteins affected by *hflKC* deletion in the WT background in LB (Fig 2A and Table 1) are highlighted. The data are for three independent cultures and can be found in S6 Data.
(EPS)

S3 Fig Changes in protein abundance in the absence of HflK or HflC proteins. (A–D) Differences in protein levels between the *ΔhflK* (A, C) and *ΔhflC* (B, D) strains and the WT, for cultures grown for 4 h at 220 rpm in LB (A, B) or TB (C, D). The data for LB are for six independent cultures; the data for TB are for three independent cultures. All data can be found in S7 Data. Proteins with differences in expression that were considered significant (see also Tables 1, S1, and S2) are labeled, with respiration-related proteins highlighted in either blue (downregulated) or red (upregulated).
(EPS)

S4 Fig Oxygen consumption by *ΔhflKC* and WT strains. A different biological replica of the experiment shown in Fig 3E. *ΔhflKC* and WT were grown in TB at 220 rpm, resuspended in fresh TB, and changes in the levels of dissolved oxygen were quantified. The lines represent the average of eight independent measurements for one culture, with error bars indicating SD. *p < 0.05, **p < 0.01 by unpaired *t* test. The data can be found in S8 Data.
(EPS)

S5 Fig Levels of ROS in WT and *ΔhflKC* cells grown in TB at 220 rpm. Cells were grown until the early exponential phase and biomass was adjusted to OD_600_ equal to 0.4 in 1 mL. DCF probe was added to the samples following incubation for 30 min in the dark. Fluorescence (485nm) was analyzed via flow cytometry. Treatment with hydrogen peroxide (H_2_O_2_) was used as a positive control for elevated ROS levels. The figure is an illustration of one representative measurement. The data can be found in S9 Data.
(EPS)

S6 Fig Membrane potential in WT and *ΔhflKC* cells grown in TB at 220 rpm. (A, B) Cells were grown until the early exponential phase and biomass was adjusted to OD_600_ equal to 0.4 in 1 mL. DiOC_2_(3) probe was added to the samples following incubation for 15 min in the dark. Fluorescence in the red (A) and green (B) was analyzed via flow cytometry. The protonophore DNP that dissipates the proton gradient across the cytoplasmic membrane was used as a control. The figure is an illustration of one representative measurement. The data can be found in S10 Data.
(EPS)

S7 Fig Impact of IspG expression on oxygen consumption and growth. (A) A different biological replicate of the experiment shown in Fig 4C. Oxygen consumption by the *ΔhflKC* strain carrying the IspG expression construct pMI107. Cells were grown in TB medium at 220 rpm, with either no or 0.02% L-arabinose induction, as indicated. The lines represent the average of eight measurements for one biological replicate. Error bars indicate the SD. ***p < 0.001 by unpaired *t* test. **(B–E)** Growth of *E. coli* YYdCas9 (WT*) strain carrying either the control pgRNA vector or pgRNA *ispG* (pMI112). To assess reduced levels of IspG, dCas9 was induced by adding 0.2 µM of anhydrotetracycline (aTC) when indicated. Cells were grown in TB medium at 100 rpm (B), 220 rpm (C), and 300 rpm (D), final OD_600_ after 8 h of growth at indicated shaking rates (E). The data represent the mean value and SD for three independent cultures. **(F)** Phase-contrast images of indicated strains after 6 h of grow in TB at 220 rpm. Scale bar is 2 µm. **(G)** Difference in protein levels between WT * carrying either pMI112 or pgRNA vector. The data are from three independent cultures. Proteins whose levels were considered to be significantly different between the two strains are labeled as in Fig 2. See also S4 Table. **(H)** Commonalities and differences between proteins that are significantly up- or downregulated during growth in TB upon *hlfKC* deletion (Fig 2B) and upon *ispG* knockdown. Labels are as in Fig 2D. All data underlying this Figure can be found in S11 Data.
(EPS)

S8 Fig Transcript level of IspG and stability analysis of proteins in *ΔhflKC* and WT strains. (A) Quantification of ispG transcript level in *ΔhflKC* and WT strains using RT-PCR. The relative mRNA level of *ispG* is quantified as the Cq value and normalized to the Cq value for the housekeeping gene *ssrA*. The data represent the mean and SD for three independent RNA samples with quadruplicate measurements each. See also S7 Table. **(B)** Abundance of indicated proteins determined by proteomics (represented as log_2_ protein intensity) in WT or *ΔhflKC* cultures grown in LB at 220 rpm, before or 30 and 60 min after inhibition of translation. The data represent the mean and SD of 18 independent cultures, measured in three different experiments with six cultures each (same dataset as in Fig 4G–4J). All data underlying this Figure can be found in S12 Data.
(EPS)

S9 Fig Sensitivity of *E. coli* growth to iron chelator. (A, B) Growth of WT and *ΔhflKC* strains in LB medium at 220 rpm, quantified by optical density at 600 nm (OD_600_). Where indicated, 20 µM of the iron chelator deferoxamine (DFO) was added to the medium. The data represent the mean and SD of three independent cultures grown in the same representative experiment and can be found in S13 Data. Indicated differences between samples: **p < 0.01, ***p < 0.001 by unpaired *t* test.
(EPS)

S10 Fig Changes in the abundance of respiratory proteins are caused by activation of the ArcAB system. (A**–**D) Growth of the WT, *ΔhflKC*, *ΔarcB*, and *ΔhflKC* *ΔarcB* strains in TB medium in a rotary shaker at 100 rpm (A), 220 rpm (B), and 300 rpm (C). Data represent the mean of three independent cultures (±SD). Final OD_600_ after 8 h of growth (D). The data represent the mean of six independent cultures (±SD). **(E,F)** Difference in protein levels between *ΔarcB* and WT strains (E). Data are for three independent cultures. Proteins whose levels were considered to be significantly different between the two strains are labeled as in Fig 2. See also S5 Table. Commonalities and differences between proteins that are significantly up- or downregulated during growth in TB upon *hflKC* deletion (Fig 2B) and upon *arcB* deletion but the sign of changes upon *arcB* deletion is inverted (F). **(G, H)** Difference in protein levels between the *ΔhflKC ΔarcB* and *ΔarcB* strains (G). The data are for three independent cultures. Labels are as in Fig 2. See also S6 Table. Commonalities and differences between proteins that are significantly up- or downregulated during growth in TB upon *hflKC* deletion (Fig 2B) and upon *ΔhflKC ΔarcB* (H). Labels are as in Fig 2D. **(I)** Quantification of *arcB* transcript level in *ΔhflKC* and WT cultures using RT-PCR. The relative mRNA level of *arcB* is quantified as the Cq value and normalized to the Cq value for the housekeeping gene *ssrA*. Data represent the mean and SD for three independent RNA samples with quadruplicate measurements each. **(J)** Abundance of ArcB and ArcA determined by proteomics in WT or *ΔhflKC* cultures grown in LB at 220 rpm, represented as log_2_ protein intensity. The data represent the mean and SD of 18 independent cultures, measured in three different experiments with six cultures each (same dataset as in Fig 4G–4J). Significance of indicated differences between samples: ns =  not significant by unpaired *t* test. All data underlying this Figure can be found in S14 Data.
(EPS)

S11 Fig Possible mechanisms of *E. coli* respiration dependence on the presence of the HflKC complex. Schematic representation of possible effects of the HflKC complex on respiration. The absence of the protective cage provided by HflKC complex might enhance proteolytic degradation of TonB by FtsH. The degradation of the iron-sulfur cluster protein IspG might be enhanced directly, or indirectly due to the reduced availability of iron that is transported by TonB. Lack of IspG, and possibly also UbiE, results in lower levels of ubiquinone-8 (CoQ_8_) and consequently abolishes the repression of the ArcB two-component sensory kinase. Activated ArcB phosphorylates the response regulator ArcA, resulting in repression of the cytochrome ubiquinol oxidase *bo*_*3*_ (Cyo) and upregulation of cytochrome ubiquinol oxidase *bd* (Cyd), as well as changes in the expression of other respiratory genes. Together, reduced levels of ubiquinone-8 and misregulation of respiratory enzymes cause the defects in respiration and growth at high oxygen levels.
(EPS)

S1 Table Additional proteins showing significant differences between *ΔhflKC*, *ΔhflK*, or *ΔhflC*, and WT strains during growth in LB at 220 rpm. (XLSX)

S2 Table Proteins showing significant differences between *ΔhflKC*, *ΔhflK*, or *ΔhflC*, and WT strains during growth in TB at 220 rpm. (XLSX)

S3 Table Proteins showing significant differences between *ΔhflKC* and WT strains during anaerobic growth in TB at 220 rpm. (XLSX)

S4 Table Proteins showing significant differences between *ispG* knockdown and WT * strains during growth in TB at 220 rpm. (XLSX)

S5 Table Proteins showing significant differences between *ΔarcB* and WT strains during growth in TB at 220 rpm. (XLSX)

S6 Table Proteins showing significant differences between *ΔhflKC ΔarcB* and *ΔarcB* strains during growth in TB at 220 rpm. (XLSX)

S7 Table Transcript levels of *ispG* and *ssrA* in *ΔhflKC* and WT strains during growth in LB at 220 rpm. (XLSX)

S8 Table Strain and plasmid list. (XLSX)

S1 Data Source data. (XLSX)

S2 Data Source data. (XLSX)

S3 Data Source data. (XLSX)

S4 Data Source data. (XLSX)

S5 Data Source data. (XLSX)

S6 Data Source data. (XLSX)

S7 Data Source data. (XLSX)

S8 Data Source data. (XLSX)

S9 Data Source data. (XLSX)

S10 Data Source data. (XLSX)

S11 Data Source data. (XLSX)

S12 Data Source data. (XLSX)

S13 Data Source data. (XLSX)

S14 Data Source data. (XLSX)

## Abbreviations

CD: Compound Discoverer
CFUs: colony-forming units
DCF: dichlorodihydrofluorescein
DFO: deferoxamine
DIA: data-independent acquisition
DNP: dinitrophenol
FRP: FLP-FLP recombination target
HCD: high-energy collision dissociation
LB: Luria-Bertani
MEP: methylerythritol phosphate
MP: membrane potential
PBS: phosphate-buffered saline
PHB: prohibitin homology
ROS: reactive oxygen species
RT-PCR: real-time PCR
SD: standard deviation
SLS: sodium-lauroyl sarcosinate
TB: tryptone broth
WT: wild-type
